# Endovascular treatment in Behçet’s disease: an integrative review

**DOI:** 10.1590/1677-5449.202200131

**Published:** 2022-06-27

**Authors:** Lígia Sant’Ana Dumont, Renan Rodrigues de Oliveira Cunha, Thais Carolina Alves Cardoso, Lygia Gomes Fleury, Augusto Wagner Santos Nunes, Pablo Ricardo França Oliveira, Hígor Chagas Cardoso

**Affiliations:** 1 Universidade Evangélica de Goiás – UniEVANGÉLICA, Anápolis, GO, Brasil.

**Keywords:** Behçet’s disease, endovascular procedures in aneurysms, vasculitis in vessels of all calibers

## Abstract

Behçet’s Disease (BD) is a rare, chronic, relapsing, inflammatory, and multisystemic disease. There is no universally described test for confirming diagnosis, so it is made clinically, on the basis of its classic triad of signs: oral ulcers, genital ulcers, and uveitis (inflammation of the uveal tract of the eye). The aim of this study is to evaluate the importance of endovascular treatment in Behçet’s disease. The literature review conducted to achieve this objective covered 30 articles published between 2002 and 2021. Behçet’s Disease affects both the venous and arterial systems. Rupture of aneurysms is the main cause of death and thus requires treatment, which can be clinical, open surgical, or endovascular. Endovascular surgery has been gaining ground for surgical treatment of arterial aneurysms, including those related to BD, although the therapy of choice is still controversial.

## INTRODUCTION

Behçet’s disease (BD) was described in the literature for the first time in 1937 by the Turkish dermatologist Hulusi Behçet.^1^ However, many authors interested in the disease prefer the term “Behçet syndrome” to “Behçet’s disease”, because its manifestations and severity can vary considerably between patients and even in terms of the prevalence of individual manifestations in different parts of the world, in particular those related to intestinal involvement.^2^ Behçet’s disease is rare, chronic, relapsing, inflammatory, and multisystemic^3^^,^ 4 and can provoke mucocutaneous, ocular, vascular, cardiac, neural, and gastrointestinal manifestations.^5^

The international diagnostic criteria for BD are recurrent oral ulceration (apthous ulcerations that recur three or more times in a 12-month period) and at least two of the following: recurrent genital ulceration (apthous ulcer or scabs), ocular lesions (anterior uveitis or retinal vasculitis), cutaneous lesions (erythema nodosum, pseudofolliculitis, or papulopustular lesions), and pathergy (skin hyperreactivity test in response to a minimal trauma, with readings in 24-48 hours)^6^^,^ 7 Vascular involvement is one of the primary predictors of morbidity and mortality in BD, with a negative impact on prognosis.^8^

Behçet’s disease is considered a systemic vasculitis when vascular involvement is present. However, it cannot be classified solely according to the caliber of the vessels involved, since it can affect small, medium, and large caliber vessels, in contrast to other types of systemic vasculitis, such as Takayasu’s arteritis and giant-cell arteritis (which have a predilection for large-caliber vessels), polyarteritis nodosa and Kawasaki disease (with predilection for medium-caliber vessels), or antineutrophil cytoplasmic antibodies vasculitis (with a preference for small-caliber vessels).^9^

The vascular form of the disease, vasculo-Behçet, is present in around 7 to 38% of cases and venous involvement is more common than arterial.^3^^,^ 10 Arterial injuries have prevalence ranging from 3.6 to 31%,^3^ generally develop in the aorta and pulmonary artery and their main branches,^11^ and can vary from aneurysms to acute arterial thromboses or stenoses, most often affecting the renal, pulmonary, and abdominal arteries.^3^ An aneurysm tends to be present in 65% of patients with arterial injury and occlusion is found in 35%.^11^ The most common manifestation is deep venous thrombosis in lower limbs. Aortic involvement is one of the most serious manifestations and is associated with high mortality rates.^3^ The mechanism of thrombosis in BD is not yet known, although antiphospholipid antibodies have been reported in some studies, but their association with BD is unclear.^12^

Occlusive arterial disease and formation of aneurysms both occur as manifestations of BD, but arterial aneurysms occur with greater frequency than occlusive disease. Incidence can be less than 5%. Arterial disease generally manifests from 3 to 8 years after the initial diagnosis, with mean age at onset of 30 years.^13^

The pathophysiology of BD has not yet been clearly defined, although it is known to be autoimmune in nature,^14^ involving the antigens HLA-B51 and HLA-B27^3^ and interaction between genetic and environmental factors, such as infection by bacteria of the genus Streptococcus,^14^ infection by the herpes simplex virus, and family history. It is thus believed that there is an abnormal immunopathological process capable of injuring and inflaming the vascular system, leading to occlusions of the circulation and to aneurysms.^10^ As a result, BD is a clinical condition that is associated with serious complications and causes significant morbidity and mortality.^15^

Epidemiologically, BD has greatest prevalence among young adults aged 20 to 40 years, with peak incidence at 30 years of age, and affects both females and males, but the condition is more severe in males.^16^ Although uncommon, it can occur in children. The fact that it presents with nonspecific initial manifestations means that it may not by recognized by pediatricians and therefore recurrent oral ulcers at any phase of childhood or adolescence should be taken as a warning of the possibility of BD.^17^

The disease has a very distinct geographical distribution, occurring predominantly in regions along the ancient “Silk Road”,^9^ running between Asia and the Mediterranean Basin, and it therefore affects many inhabitants of Japan, Iran, and Turkey. Prevalence is low in Western countries. For example, it does not exceed 0.6 cases per 100,000 inhabitants in the United States.^5^ In contrast, prevalence varies from 17 to 20 cases per 10,000 inhabitants in oriental countries. There are few epidemiological data on vasculites in Brazil, but those that are available suggest that BD is the country’s most common systemic vasculitis, followed by Takayasu’s Arteritis.^9^

There is no universally accepted test to diagnose BD, so diagnosis is made clinically, on the basis of its classic triad of signs: oral ulcers, genital ulcers, and uveitis.^3^ Symptoms occur in a pattern of unexpected exacerbations and remissions and the frequency of episodes reduces over time.^18^ The International Study Group (ISG) created the International Criteria for Behçet’s Disease (ICBD) in order to facilitate diagnosis of the disease. A diagnosis of BD according to the ICBD requires four points or more, scoring two points each for ocular lesions, oral aphthosis, and genital aphthosis and one point each for skin lesions, central nervous system (CNS) involvement, cutaneous manifestations, and a positive pathergy test.^5^

Prognosis varies from one BD patient to another, but the disease can be severe. The mucocutaneous damage can be very debilitating and can have profound effects on patients’ quality of life. The ocular damage compromises visual acuity, with a non-negligible risk of blindness. The neurological damages expose patients to a risk of serious neurological sequelae. Finally, the vascular damage is severe, arterial injuries particularly so, and remains the major cause of death of patients with BD.^19^

The objective of treatment is prevention of irreversible organ damage, especially in the early and active phase of BD, targeting the clinical manifestations, and management is dependent on the severity of involvement of the organ in question.^5^ Antagonists of tumor necrosis factor alpha (TNF-α) and interferon (IFN) have demonstrated good efficacy and are the first-line agents used to improve BD prognosis. However, data on these ideal therapeutic approaches are still scarce and there is a lack of informative laboratory biomarkers to monitor disease progression.^10^

With regard to the disease’s vascular manifestations, aneurysms are rare, but since aneurysm rupture is the principal cause of death from this disease, they should be treated as soon as possible after identification.^3^ Aneurysms in BD patients are treated whenever possible, because of the high risk of rupture,^20^ but this is the only situation in which anticoagulants should not be used.^21^ Treatment of aneurysms secondary to BD can be accomplished in three ways: clinical, open surgical, or endovascular. Clinical treatment has been described above, while surgical correction of aortic aneurysms can be achieved in two different ways: by resection and substitution with a prosthetic graft or by aneurysmectomy with direct closure with a pericardial patch, for saccular aneurysms.^3^ The main advantages of endovascular treatment are lower mortality rates, such as 0.6 to 3.5%, even in high risk groups, and higher success rates (97%). This alternative method can yield a shorter length of hospital stay and a significantly shorter period before returning to normal life. General anesthesia and surgical dissection are avoided to reduce morbidity.^22^ Endovascular treatment of arterial aneurysms has proven safe and effective, with acceptable rates of vascular complications and excellent patency of the site treated.^21^ The objective of this study is therefore to summarize and analyze information and experiences reported in articles on the importance of endovascular treatment in BD.

## MATERIALS AND METHODS

This integrative review of the literature is based on 30 articles, 19 of which covered endovascular treatment in BD. Searches were conducted for articles that related BD to endovascular treatment. The research question was: How important is endovascular treatment in BD? In order to answer this question, searches were run on the following databases: Scientific Electronic Library Online (SciELO), Google Scholar, and Publisher Medline (PubMed). Selection criteria were articles published from 2002 to 2021 in Portuguese or English. The Health Sciences Descriptors (DeCS) keywords used were as follows: “Síndrome de Behçet”, “Procedimentos Endovasculares” and “Terapêutica”. The Medical Subject Headings (MeSH) “Behçet Syndrome”, “Endovascular Procedures”, and “Therapeutics” were also used. Inclusion criteria for the studies were articles with full text available published in Portuguese or English. All of the studies selected were published from 2002 onwards. After reading and analysis of the content of the articles chosen, a table was constructed to summarize all of the endovascular treatments covered in relation to BD (Table 1).

**Table 1:** Articles about use of endovascular treatment in Behçet’s disease.

**Year**	**Authors**	**Evidence level**	**Study**	**Endovascular treatment**
2021	Metzger, PB; Costa, KR; Metzger, SL; Almeida, LC.^3^	3b	Hospital Geral Roberto Santos (HGRS), Salvador, Bahia, Brazil.	Endovascular repair of abdominal aortic aneurysm complicated by Adamantiades-Behçet disease
2019	Belczak, SQ; Silva, IT; Marques, GG; Copetti, LF; Stefaniak, V; Quintas, GG; Uchimura, KB.^23^	3b	Instituto de Aprimoramento e Pesquisa em Angiorradiologia e Cirurgia Endovascular (IAPACE), São Paulo, São Paulo, Brazil.	Endovascular intervention in patients with Behçet’s disease with arterial aneurysms
2018	Lemos, LBF.^8^	3b	Instituto de Ciências Biomédicas Abel Salazar (ICBAS-UP), Universidade do Porto, Porto, Portugal.	Vascular involvement in Behçet’s disease
2017	Castro Júnior, DF; Soares, LP; Frazão, CTV; Frazão Júnior, GA; Neves, LL; Costa Júnior, AF; Silva, AS; Rossoni, HCM.^4^	3b	Centro Universitário UnirG, Gurupi, Tocantins, Brazil.	Endovascular treatment of popliteal artery aneurysm in a young patient with Behçet’s disease
2017	Souza, NLAR; Siqueira, DED; Cantador, AA; Rossetti, LP; Molinari, GJDP; Guillaumon, AT.^14^	3b	Hospital das Clínicas (HC), Universidade Estadual de Campinas (UNICAMP), Campinas, São Paulo, Brazil.	Endovascular repair of an abdominal aortic aneurysm with erosion of lumbar vertebra, associated with Behçet’s disease
2016	Lucas, ML; Frankini, T; Frankini, A; Aerts, N; Tourinho, TF.^24^	3b	Department of Medicine, Universidade Federal de Ciências da Saúde de Porto Alegre (UFCSPA), Porto Alegre, Rio Grande do Sul, Brazil.	Endovascular treatment of ruptured celiac trunk aneurysm in a patient with Behçet’s disease
2015	Camargo, PAB; Bertanha, M; Sobreira, RMML; Jaldin, RG; Yoshida, RA; Pimenta, REF; Yoshida, WB^25^|.	3b	Faculdade de Medicina de Botucatu, Universidade Estadual Paulista (UNESP), Botucatu, São Paulo, Brazil.	Endovascular treatment in patients with Behçet’s disease associated with thoracoabdominal pseudoaneurysms
2015	Detanico, AB; Brandão, ML; Fernandes, LF; Camelo, CPR; Santos, JRS.^16^	3b	Vascular surgery service at the Hospital das Clínicas da Universidade Federal de Goiás, Goiânia, Goiás, Brazil.	Endovascular treatment in patients with aortic thrombosis with late-diagnosed Behçet’s disease
2010	Belczak, SQ; Aun, R; Valentim, L; Sincos, IR; Nascimento, LD; Puech-Leão, P.^1^	3a	Hospital das Clínicas (HC), Universidade de São Paulo (USP), São Paulo, São Paulo, Brazil.	Endovascular treatment of aortic aneurysms in patients with Behçet’s disease
2010	Laurenti, MR; Demartini Junior, Z; Santos, RMT; Spotti, AR.^26^	3b	Hospital de Base e Centro do Cérebro e Coluna, São José do Rio Preto, São Paulo, Brazil.	Endovascular treatment of dissecting vertebral artery aneurysm in patients with Behçet’s disease
2009	Kim, WH; Choi, D; Kim, JS; Ko, YG; Jang, Y; Shim, WH.^20^	2b	Medical Faculty at the Yonsei University, Shinchon-dong, Seodaemun-gu, Seoul, Republic of Korea.	Efficacy and safety of endovascular aneurysm repair in patients with vasculo-Behçet disease
2008	Meyer Neto, JGC; Assuf, S; Penna, GL; Morard, MRS.^6^	3b	Hospital Federal de Ipanema, Rio de Janeiro, Rio de Janeiro, Brazil.	Endovascular treatment of large pulmonary artery aneurysm in a patient with Behçet’s disease
2007	Jung, NY; Kim, SK; Chung, EC; Park, H; Cho, YK.^11^	3b	Department of Internal Medicine, Kangbuk Samsung Hospital, Medical School, Sungkyunkwan University, Seoul, Republic of Korea.	Endovascular treatment of ruptured intrahepatic artery aneurysm in a patient with Behçet syndrome
2006	D’Alessandro, GS; Machietto, RF; Silva, SM; Campos Júnior, W; Akel, CJ; Etchebehere, RM; Cardoso, RM; Izukawa, NM.^7^	3b	Angiology and Vascular Surgery Service, Hospital Professor Edmundo Vasconcelos, São Paulo, São Paulo, Brazil.	Endovascular repair of popliteal artery aneurysm as manifestation of decompensated Behçet’s disease
2006	Serratto, VA; Loyola Netto, JG; Yoshizumi, L; Paiva, E.^12^	3b	Rheumatology Service, Internal Medicine Department, Hospital de Clínicas da Universidade Federal do Paraná (UFPR), Curitiba, Paraná, Brazil.	Endovascular treatment of Behçet’s disease with extensive venous thrombosis
2005	Alves, CMR.^27^	2a	Escola Paulista de Medicina, Universidade Federal de São Paulo, São Paulo, São Paulo, Brazil.	Endovascular treatment in special situations: connective tissue diseases, non-infectious aortites, mycotic aneurysms, isolated iliac artery aneurysms, and emergencies
2005	Robazzi, TCMV; Arruti, R; Souza, AK; Santiago, MB.^28^	3b	Pediatric rheumatology service, Hospital São Rafael, Salvador, Bahia, Brazil.	Endovascular treatment of neuro-Behçet disease with childhood onset
2004	Silistreli, E; Karabay, O; Erdal, C; Serbest, O; Guzeloglu, M; Çatalyurek, H; Açikel, U; Turkey, I.^22^	3b	Cardiovascular Surgery Department, DokuzEylül University Medical School, Izmir, Turkey.	Behçet’s disease: treatment of popliteal artery pseudoaneurysm with endovascular stent and graft.
2002	Albuquerque, PR; Terreri, MTRA; Len, CA; Hilário, MOE.^17^	2b	Escola Paulista de Medicina, São Paulo, São Paulo, Brazil.	Behçet’s disease in childhood

## RESULTS AND DISCUSSION

Behçet’s disease can involve both the venous and arterial systems, with an 88% incidence of venous thromboses/thrombophlebitis and varicose veins in those with venous involvement and 12% incidence of occlusions/stenosis, aneurysms, and pseudoaneurysms.^25^ Arterial injuries are less frequent than venous damage in those with vascular involvement and arterial lesions are responsible for just 12% of all vascular complications in Behçet syndrome. Arterial lesions generally develop in the aorta and pulmonary artery and their major branches.^9^ The most common site of aneurysm formation is the abdominal aorta, followed by the pulmonary, femoral, subclavian, popliteal, common carotid, coronary, brachial, ulnar, common iliac, external iliac, tibial, renal, cerebral, axillary, and splenic arteries,^9^ while 65% are due to aneurysmal degeneration and 35% to occlusive diseases.^25^ Behçet syndrome is now recognized as a systemic disorder with mucocutaneous, ophthalmic, neurological, cardiovascular, pulmonary, gastrointestinal, urogenital, and musculoskeletal involvement. Any artery or vein in the body can be affected, and involvement of the vascular tree manifests pathologically as arterial occlusions, aneurysms, venous occlusions, and varicose veins.^9^ While aneurysms are not very prevalent in BD, their rupture is the principal cause of death. These ruptures are related to inflammatory processes in perianeurysmal tissues and fibrotic reactions.^3^ The treatment options available are those already mentioned above: clinical, open surgical, and endovascular. Specifically in relation to endovascular treatment, since 1984 there have been increasing numbers of reports of the efficacy of this technique for treatment of abdominal aortic aneurysms (AAA) in patients with BD.^3^

Systemic vasculites constitute a group of rare and heterogeneous diseases of unknown etiology, characterized by inflammation and necrosis of the walls of vessels. The prevalence of BD is high in countries along the Silk Road (i.e. from the Middle East to the Far East, including Turkey, Iran, China, and Japan) and in Mediterranean countries.^9^

The prevalence and incidence of the systemic vasculites are still unknown in Brazil. The country has a large and heterogeneous population, which includes people of Portuguese origin, black people, and indigenous people, and very often people with mixtures of these origins.^9^ People of Italian and German descent are common in the South and Southeast of the country, while those of Japanese, Korean, Jewish, Lebanese, and Syrian descent generally live in the Southeast of Brazil, especially in the state of São Paulo. As a result, significant differences in the epidemiology of systemic vasculites can be found all over Brazil.^9^

Clinical treatment using corticosteroids and immunosuppressants (cyclophosphamide and azathioprine)^3^ should be attempted before any surgical treatment, whether open or endovascular, since there is a high rate of aneurysmal disease recurrence among BD patients.^14^ However, urgent treatment is indicated if remission of important symptoms does not occur and the risk of rupture does not recede with clinical treatment alone.^25^ There is also the possibility of adjuvant use of corticosteroids or immunosuppressants during the postoperative period and also of use of anticoagulants or platelet antiaggregants, with the objective of reducing the risk of graft occlusion.^16^

Treatment of BD targets prevention of the irreversible damage that primarily occurs during the initial course of the disease, especially in the high risk group (young men), and also aims to prevent exacerbation of mucocutaneous and joint involvement, which generally do not cause damage, but affect quality of life. All patients with BD and ocular inflammatory disease affecting the posterior segment should be put on a treatment regimen including azathioprine and systemic corticosteroids.^29^ Ocular involvement in BD follows a recurrent and relapsing course and the repeated inflammatory attacks result in irreversible damage and loss of sight. The objectives should be suppression of inflammation and prevention of recurrence of ocular attacks. There is no firm evidence to guide management of BD affecting the major vessels.^29^

Immunosuppressants such as corticosteroids, azathioprine, cyclophosphamide, or cyclosporine A are recommended for treatment of acute deep vein thrombosis in BD. Cyclophosphamide and corticosteroids are recommended for management of pulmonary and peripheral arterial aneurysms.^29^

TNF-α and IFN antagonists have shown good efficacy and are the first-line agents used to improve prognosis of BD. Treatment with TNF-α antagonists (infliximab, etanercept, and adalimumab) is based on control of the inflammatory response and has proven effective for severe and refractory manifestations of BD. Treatment with IFN, particularly INF-α, has demonstrated benefits for habitual treatment of the disease, with antiviral, antitumoral, and immunomodulatory activities that are effective in management of BD.^29^ However, data on the ideal therapeutic approaches are still scarce and there is a lack of informative laboratory markers to monitor disease progression.^10^

Mycotic aneurysms have incidence of 1% of all aneurysms and are caused by remote bacterial infections. Intravascular grafts are contraindicated when treating these false aneurysms, but successful stenting via endovascular surgery is described in the literature and reduces the risks of conventional surgery, particularly in the thoracic aorta or involving multiple segments.^27^

The abdominal aorta is the artery most commonly affected, followed by the femoral and pulmonary arteries, while intracranial aneurysms have low incidence. However, surgical treatment is the first-choice for ruptured cerebral aneurysms associated with BD, although endovascular treatment is a reasonable alternative to surgery for ruptures located peripherally, with fusiform format, for pseudoaneurysm dissections, and in the posterior circulation.^26^ Manifestations related to the CNS occur most frequently in adults. These constitute the most severe form of the disease, manifesting from 2 to 6 years after initial onset of symptomology, with prevalence from 5 to 7% in the adult population.^28^

Endovascular treatment, with placement of endoprostheses or covered stents or coil embolization, can be employed in elective situations with favorable anatomy. Diagnosis of vascular complications, primarily of aneurysms, combined with correct treatment, especially endovascular, results in better prognosis for patients.^24^ Thus, endovascular surgery has become the preferred approach for surgical treatment in cases of arterial aneurysms, especially those related to BD.^1^ Arterial aneurysms are more common and constitute an important cause of death from BD, secondary to rupture. For this reason, surgical treatment is obligatory and should be performed as soon as possible.^4^

The best treatment for aneurysms in patients with BD is still controversial.^1^ In general, treatment of BD varies according to the patient’s clinical manifestations and their severity,^30^ but endovascular treatment is a relevant option for patients classified as at high risk, since morbidity and mortality rates (0.6-3.5%) after conventional surgery remain high.^1^ Moreover, open surgical repair has less satisfactory results in terms of postoperative vascular complications, such as pseudoaneurysm and graft occlusion, and the endovascular method is thus less invasive.^1^ It is linked with a lower likelihood of complications during the postoperative period, with incidence of around 19%.^23^ Endovascular treatment is therefore proving to be a promising alternative to open surgery for treating these patients^14^ and has already become the approach of choice for the majority of patients with high surgical risk.^23^

Endovascular treatment generally involves shorter operating time, shorter hospital stay, and lower blood loss compared with open surgical treatment. Furthermore, endovascular prosthesis deployment has a high success rate (90% in low risk patients and 80% patients with moderate to high risk). On the other hand, endovascular treatment is also subject to limitations, primarily related to delivery system size, to leaks, and to the position of main trunks.^1^

## CONCLUSIONS

Although there is not yet a consensus on the best treatment for vascular aneurysms in patients with BD, endovascular surgery is becoming the preferred option over open surgical repair, particularly in patients with high surgical risk, because of its benefits in terms of operating time, length of hospital stay, and blood loss. Regardless, treatment with corticosteroids and immunosuppressants such as cyclophosphamide and azathioprine are used as adjuvant therapy both preoperatively and during the postoperative period for patients who have BD-related aneurysms repaired, irrespective of the technique chosen.

## References

[1] Belczak SQ, Aun R, Valentim L, Sincos IR, Nascimento LD, Puech-Leão P (2010). Tratamento endovascular de aneurismas de aorta em pacientes com doença de Behçet: relato de dois casos. J Vasc Bras.

[2] Barnes CG (2006). Treatment of Behçet’s syndrome. Rheumatology.

[3] Metzger PB, Costa KR, Metzger SL, Almeida LC (2021). Tratamento endovascular de aneurisma sacular aórtico associado à doença de Adamantiades-Behçet. J Vasc Bras.

[4] Castro DF, Soares LP, Frazão CTV (2017). Aneurisma de artéria poplítea em paciente jovem com doença de Behçet. Relatos Casos Cir..

[5] Ferrão C, Almeida I, Marinho A (2015). A nossa regra de ouro na doença de Behçet: tratar a manifestação clínica. Arq Med.

[6] Meyer JGC, Assuf S, Penna GL, Morard MRS (2008). Aneurisma volumoso da artéria pulmonar em paciente portador de Doença de Behçet. Relato de Caso. Rev Bras Clin Med..

[7] D’Alessandro GS, Machietto RF, Silva SM (2006). Aneurisma de artéria poplítea como manifestação da doença de Behçet descompensada. J Vasc Bras.

[8] Lemos LBF (2018). Envolvimento vascular na doença de Behçet.

[9] Belem JMFM, Pereira RSR, Perez MO (2020). Epidemiologic features of systemic vasculitides in the southeast region of Brazil: hospital-based survey. J Clin Rheumatol.

[10] Vargas RM, Cruz MLN, Giarllarielli MPH (2021). Acometimento vascular da doença de Behçet: o processo imunopatológico. J Vasc Bras.

[11] Jung NY, Kim SK, Chung EC, Park H, Cho YK (2007). Endovascular treatment for rupture of intrahepatic artery aneurysm in a patient with Behçet’s Syndrome. AJR.

[12] Serratto VA, Loyola JG, Yoshizumi L, Paiva E (2006). Doença de Behçet com extensa trombose venosa. Rev Bras Reumatol.

[13] Morata AR, Conde AH, Cosme CC (2012). Atypical vascular involvement in a case of Behçet’s disease..

[14] Souza NLAR, Siqueira DED, Cantador AA, Rossetti LP, Molinari GJDP, Guillaumon AT (2017). Tratamento endovascular de aneurisma de aorta abdominal com erosão de vértebra lombar associada à doença de Behçet: relato de caso. J Vasc Bras.

[15] Cruz BA (2005). Atualização em Doença de Behçet. Rev Bras Reumatol.

[16] Detanico AB, Brandão ML, Fernandes LF, Camelo CPR, Santos JRS (2015). Trombose aórtica em paciente com diagnóstico tardio de Doença de Behçet. J Vasc Bras.

[17] Albuquerque PR, Terreri MTRA, Len CA, Hilário MOE (2002). Doença de Behçet na infância. J Pediatr (Rio J).

[18] Oliveira MRC (2015). Doença de Behçet: aspectos importantes e característicos.

[19] Kone-Paut I, Barete S, Bodaghi B (2021). French recommendations for the management of Behçet’s disease. Orphanet J Rare Dis.

[20] Kim WH, Choi D, Kim JS, Ko YG, Jang Y, Shim WH (2009). Effectiveness and safety of endovascular aneurysm treatment in patients with Vasculo-Behçet Disease. J Endovasc Ther.

[21] Collins TR (2016). 2015 ACR/ARHP Annual Meeting: Behçet’s Disease Poses Diagnosis, Treatment Challenges.

[22] Silistreli E, Karabay O, Erdal C (2004). Behçet’s disease: treatment of popliteal pseudoaneurysm by an endovascular stent graft implantation. Ann Vasc Surg.

[23] Belczak SQ, Silva IT, Marques GG (2019). Tratamento endovascular da doença de Behçet: relato de caso. J Vasc Bras.

[24] Lucas ML, Frankini T, Frankini Â, Aerts N, Tourinho TF (2016). Ruptura de aneurisma de tronco celíaco em paciente com doença de Behçet. Rev Col Bras Cir.

[25] Camargo PAB, Bertanha A, Moura R (2015). Correção endovascular de pseudoaneurisma toracoabdominal em paciente com Doença de Behçet. J Vasc Bras.

[26] Laurenti MR, Demartini Z, Santos RMT, Spotti AR (2010). Dissecting aneurysm of the vertebral artery and Behçet’s disease. J Bras Neurocirurg..

[27] Alves CMR (2005). Tratamento endovascular em situações especiais: doença do tecido conectivo, aortites não-infecciosas, aneurismas micóticos, aneurisma isolado das artérias ilíacas e urgências. Rev Bras Cardiol Invas..

[28] Robazzi TCMV, Arruti R, Souza AK, Santiago MB (2005). Doença de Neuro-Behçet de início na infância. Rev Bras Reumatol.

[29] Hatemi G, Silman A, Bang D (2008). EULAR recommendations for the management of Behçet disease. Ann Rheum Dis.

[30] Silva OF, Araújo RHS, Freire EAM (2006). Doença de Behçet cursando com trombose de veia cava superior. J Vasc Bras.

