# Supplementary material for: Endovascular treatment in Behçet’s disease: an integrative review
Source: J Vasc Bras. 2022 Jun 27;21:e20220013. doi: 10.1590/1677-5449.202200131 (PMC9242428; doi:10.1590/1677-5449.202200131)
Supplement: Translation

# Translation: Tratamento endovascular na doença de Behçet: uma revisão integrativa

**DOI:** 10.1590/1677-5449.202200132

**Authors:** Lígia Sant’Ana Dumont, Renan Rodrigues de Oliveira Cunha, Thais Carolina Alves Cardoso, Lygia Gomes Fleury, Augusto Wagner Santos Nunes, Pablo Ricardo França Oliveira, Hígor Chagas Cardoso

**Affiliations:** 1 Universidade Evangélica de Goiás – UniEVANGÉLICA, Anápolis, GO, Brasil.

**Keywords:** doença de Behçet, procedimentos endovasculares em aneurismas, vasculites de vasos de todos os calibres

## Abstract

A doença de Behçet (DB) é caracterizada por ser uma doença rara, crônica, recidivante, inflamatória e multissistêmica. Quanto ao diagnóstico, não existe nenhum teste universalmente descrito; logo, ele é feito de maneira clínica, por meio da presença da sua tríade clássica de sinais: úlceras orais, úlceras genitais e uveíte (inflamação do trato uveal do olho). O objetivo deste estudo foi avaliar a importância do tratamento endovascular na doença de Behçet, sendo que a revisão de literatura para obter essa resposta foi realizada a partir de 30 artigos publicados entre 2002 e 2021. A DB acomete tanto o sistema venoso quanto o arterial. A ruptura de aneurismas é a principal causa de morte e necessita de tratamento, podendo ser clínico, cirúrgico aberto ou por via endovascular. A cirurgia endovascular vem ganhando espaço no tratamento cirúrgico nos casos de aneurismas arteriais, inclusive nos relacionados com DB, apesar de a terapia de escolha ainda ser controversa.

## INTRODUÇÃO

A doença de Behçet (DB) foi descrita pela primeira vez na literatura em 1937 pelo médico dermatologista Hulusi Behçet, de origem turca^1^. Entretanto, o uso do nome “síndrome de Behçet”, em vez de “doença de Behçet”, é preferido por muitos dos interessados ​​na doença, pois pode apresentar variações consideráveis ​​nas manifestações e gravidade entre os pacientes e, de fato, na prevalência de manifestações individuais em diferentes partes do mundo, especialmente o envolvimento intestinal^2^. Ela é caracterizada por ser rara, crônica, recidivante, inflamatória e multissistêmica^3^^,^ 4, que pode cursar com manifestações mucocutâneas, oculares, vasculares, cardíacas, nervosas e gastrointestinais^5^.

Os critérios diagnósticos internacionais da doença são ulceração oral recorrente (ulcerações aftosas que recorrem três vezes ou mais em um período de 12 meses) e mais dois dos seguintes: ulceração genital recorrente (úlcera aftosa ou escaras), lesões oculares (uveíte anterior ou vasculite retiniana), lesões cutâneas (eritema nodoso, pseudofoliculite, lesões papulopustulosas) e patergia (teste de hiper-reatividade cutânea em resposta a um trauma mínimo, com leituras em 24-48 horas)^6^^,^ 7. O envolvimento vascular é um dos principais preditores de morbidade e mortalidade da doença, afetando negativamente o prognóstico^8^.

Quando há esse envolvimento vascular, a DB é considerada uma vasculite sistêmica. No entanto, ela não pode ser classificada isoladamente de acordo com o calibre do vaso acometido, tendo em vista que pode acometer vasos de pequeno, médio ou grande calibre, diferentemente de outras vasculites sistêmicas, como a arterite de Takayasu e a arterite de células gigantes, que têm predileção por vasos de grande calibre, a poliarterite nodosa e a doença de Kawasaki, com predileção por vasos de médio calibre, e as vasculites associadas aos anticorpos anticitoplasma de neutrófilos, com predileção por vasos de pequeno calibre^9^.

A forma vascular da doença, a vásculo-Behçet, está presente em cerca de 7 a 38% dos casos, sendo o envolvimento venoso mais comum que o arterial^3^^,^ 10. As lesões arteriais têm uma prevalência entre 3,6 e 31%^3^, geralmente se desenvolvem na aorta e na artéria pulmonar e em seus principais ramos^11^ e podem variar desde aneurismas até estenoses ou tromboses arteriais agudas, sendo as artérias renais, pulmonares e abdominais as mais afetadas^3^. Um aneurisma costuma estar presente em 65% dos pacientes com lesão arterial e a oclusão, em 35%^11^. A principal manifestação é a trombose venosa profunda de membros inferiores. O envolvimento aórtico é uma das manifestações mais severas e está associado a altas taxas de mortalidade^3^. O mecanismo de trombose na DB é ainda desconhecido; entretanto, a presença de anticorpos antifosfolípides tem sido relatada em alguns estudos, mas não está clara a sua associação com a DB^12^.

Tanto a doença arterial oclusiva quanto a formação de aneurisma ocorrem como manifestações da DB, mas os aneurismas arteriais ocorrem com mais frequência do que a doença oclusiva. A incidência pode ser inferior a 5%. A doença arterial geralmente se manifesta de 3 a 8 anos após o diagnóstico inicial, com média de idade de 30 anos^13^.

Em relação à fisiopatologia da DB, ela ainda não é bem definida; no entanto, sabe-se que é de natureza autoimune^14^, em que há o envolvimento dos antígenos HLA-B51 e HLA-B27^3^ e interação entre fatores genéticos e ambientais, como a infecção por bactérias do gênero Streptococcus^14^, infecção pelo vírus do herpes simples e história familiar. Nesse sentido, acredita-se que haja um processo imunopatológico anormal capaz de lesionar e inflamar o sistema vascular, o que leva a oclusões na circulação e aneurismas^10^. Dessa forma, a DB mantém-se como uma condição clínica associada a complicações graves, com morbimortalidade expressiva^15^.

Acerca da epidemiologia, a DB tem maior prevalência em adultos jovens entre 20 e 40 anos, com pico de incidência aos 30 anos, e acomete tanto o sexo feminino quanto o masculino, mas a condição no sexo masculino é mais severa^16^. Apesar de ser pouco frequente, pode ocorrer na infância. Pelo fato de apresentar manifestações iniciais inespecíficas, pode não ser reconhecida pelo pediatra; portanto, úlceras orais recorrentes em qualquer fase da infância e adolescência devem alertar para a possibilidade do diagnóstico de DB^17^.

Sua distribuição geográfica é muito peculiar, predominando nas regiões da antiga “Rota da Seda”^9^, localizada entre a Ásia e a Bacia do Mediterrâneo, atingindo, assim, muitos habitantes do Japão, do Irã e da Turquia. Sua prevalência nos países ocidentais é baixa, por exemplo, nos Estados Unidos da América, ela não ultrapassa 0,6 casos a cada 100.000 habitantes^5^. Em países orientais, a prevalência varia de 17 a 20 casos a cada 10.000 habitantes. Nesse contexto, há poucos dados epidemiológicos sobre vasculites no Brasil, e os existentes apontam para que a DB seja a vasculite sistêmica mais frequente, seguida da arterite de Takayasu^9^.

No que tange ao diagnóstico, não existe nenhum teste universalmente aceito para o diagnóstico de DB, sendo ele clínico e realizado por meio da presença de sua tríade clássica: úlceras orais, úlceras genitais e uveíte^3^. A ocorrência dos sintomas se caracteriza por remissões e exacerbações inesperadas, e, com o passar do tempo, ocorre uma diminuição da frequência dos episódios^18^. Na tentativa de ajudar no diagnóstico da doença, o International Study Group (ISG) deu origem ao International Criteria for Behçet’s Disease (ICBD). No ICBD, o diagnóstico de DB exige quatro ou mais pontos, atribuindo dois pontos às lesões oculares, à aftose oral e à aftose genital, enquanto as lesões da pele, o envolvimento do sistema nervoso central (SNC), as manifestações cutâneas e o teste de patergia positivo contam um ponto cada^5^.

Em relação ao prognóstico da DB, é variável de um paciente para outro, mas a doença pode ser grave. O dano mucocutâneo pode ser muito debilitante e afetar profundamente a qualidade de vida do paciente. O dano ocular compromete a acuidade visual, com um risco não negligenciável de cegueira. O dano neurológico acarreta a exposição ao risco de sequelas neurológicas graves. Finalmente, o dano vascular, principalmente o arterial, é grave e continua sendo a principal causa de morte em pacientes acometidos pela DB^19^.

O objetivo do tratamento perpassa à prevenção do dano irreversível do órgão, especialmente na fase precoce e ativa da DB, sendo dirigido à manifestação clínica, sendo que o tratamento desses pacientes depende da gravidade do envolvimento de órgão em causa^5^. Os antagonistas do fator de necrose tumoral alfa (TNF-α) e do interferon (IFN) têm demonstrado boa eficácia e são os agentes de primeira linha usados para melhorar o prognóstico da DB. No entanto, ainda há escassez de dados sobre essas abordagens terapêuticas ideais, e há falta de marcadores laboratoriais informativos para monitorar a progressão da doença^10^.

Em relação às manifestações vasculares, os aneurismas são raros. No entanto, a sua ruptura constitui a principal causa de morte nessa doença, o que justifica o tratamento mais precoce possível após a identificação^3^. Na DB, o tratamento do aneurisma é realizado sempre que possível devido ao alto risco de ruptura^20^, mas esse é o único cenário em que os anticoagulantes não devem ser usados^21^. O tratamento do aneurisma secundário à DB pode ser realizado de três formas: clínico, cirúrgico aberto ou via endovascular. O tratamento clínico consiste naquele já citado acima, enquanto a correção cirúrgica do aneurisma de aorta pode ser feita de duas formas distintas: por meio da ressecção e substituição com prótese ou por aneurismectomia com fechamento direto com uso de *patch* de pericárdio em aneurismas saculares^3^. As principais vantagens do tratamento endovascular são níveis de mortalidade mais baixos, como 0,6 a 3,5% mesmo em grupos de alto risco, e maiores taxas de sucesso (97%). Esse método alternativo pode resultar em menor tempo de internação e significativamente menor período até o retorno à vida normal. A anestesia geral e a dissecção cirúrgica são evitadas, para que se tenha diminuição da morbidade^22^. O tratamento endovascular dos aneurismas arteriais se mostrou eficaz e seguro, com taxa aceitável de complicações vasculares e excelente perviedade do local tratado^21^. Com isso, este estudo tem como objetivo sintetizar e analisar as informações e experiências obtidas por meio de artigos redigidos a respeito da importância do tratamento endovascular na DB.

## MATERIAIS E MÉTODOS

A construção desta revisão integrativa de literatura foi realizada a partir de 30 artigos, em que 19 abordam o tratamento endovascular na DB. Foram realizadas pesquisas de artigos que relacionassem a DB com o tratamento endovascular. A questão norteadora do estudo foi: qual a importância do tratamento endovascular na DB? Para responder a tal questionamento, foi executada uma busca nas seguintes bases de dados: Scientific Electronic Library Online (SciELO), Google Acadêmico e Publisher Medline (PubMed). Para isso, foram selecionados artigos publicados entre 2002 e 2021, nas línguas portuguesa e inglesa. Os Descritores em Ciências da Saúde (DeCS) utilizados foram os seguintes: “Síndrome de Behçet”, “Procedimentos Endovasculares” e “Terapêutica”. Além destes, foram utilizados Medical Subject Headings (MeSH): “*Behçet Syndrome*”, “*Endovascular Procedures*” e “*Therapeutics*”. Os critérios de inclusão dos estudos foram artigos disponíveis com texto completo e estudos publicados nos idiomas português e inglês. Todos os trabalhos escolhidos foram a partir de 2002. Após a leitura e análise de conteúdo dos artigos selecionados, foi possível a construção de um quadro que sintetiza todos os tratamentos endovasculares abordados na DB, apresentados na Tabela 1.

**Tabela 1:** Artigos que abordam o uso do tratamento endovascular na doença de Behçet.

**Ano**	**Autores**	**Grau de evidência**	**Local de estudo**	**Tratamento endovascular**
2021	Metzger, PB; Costa, KR; Metzger, SL; Almeida, LC^3^.	3b	Hospital Geral Roberto Santos (HGRS), Salvador, Bahia, Brasil.	Tratamento endovascular do aneurisma de aorta abdominal complicado pela doença de Adamantiades-Behçet
2019	Belczak, SQ; Silva, IT; Marques, GG; Copetti, LF; Stefaniak, V; Quintas, GG; Uchimura, KB^23^.	3b	Instituto de Aprimoramento e Pesquisa em Angiorradiologia e Cirurgia Endovascular (IAPACE), São Paulo, São Paulo, Brasil.	Intervenção endovascular em pacientes portadores de doença de Behçet com aneurismas arteriais
2018	Lemos, LBF^8^.	3b	Instituto de Ciências Biomédicas Abel Salazar (ICBAS-UP), Universidade do Porto, Porto, Portugal.	Envolvimento vascular na doença de Behçet
2017	Castro Júnior, DF; Soares, LP; Frazão, CTV; Frazão Júnior, GA; Neves, LL; Costa Júnior, AF; Silva, AS; Rossoni, HCM^4^.	3b	Centro Universitário UnirG, Gurupi, Tocantins, Brasil.	Tratamento endovascular em aneurisma de artéria poplítea em paciente jovem com doença de Behçet
2017	Souza, NLAR; Siqueira, DED; Cantador, AA; Rossetti, LP; Molinari, GJDP; Guillaumon, AT^14^.	3b	Hospital das Clínicas (HC), Universidade Estadual de Campinas (UNICAMP), Campinas, São Paulo, Brasil.	Tratamento endovascular de aneurisma de aorta abdominal com erosão de vértebra lombar associada à doença de Behçet
2016	Lucas, ML; Frankini, T; Frankini, A; Aerts, N; Tourinho, TF^24^.	3b	Curso de Medicina da Universidade Federal de Ciências da Saúde de Porto Alegre (UFCSPA), Porto Alegre, Rio Grande do Sul, Brasil.	Tratamento endovascular em ruptura de aneurisma de tronco celíaco em paciente com doença de Behçet
2015	Camargo, PAB; Bertanha, M; Sobreira, RMML; Jaldin, RG; Yoshida, RA; Pimenta, REF; Yoshida, WB^25^.	3b	Faculdade de Medicina de Botucatu, Universidade Estadual Paulista (UNESP), Botucatu, São Paulo, Brasil.	Tratamento endovascular em pacientes com doença de Behçet associado com pseudoaneurisma toracoabdominal
2015	Detanico, AB; Brandão, ML; Fernandes, LF; Camelo, CPR; Santos, JRS^16^.	3b	Serviço de Cirurgia Vascular do Hospital das Clínicas da Universidade Federal de Goiás, Goiânia, Goiás, Brasil.	Tratamento endovascular em pacientes com trombose aórtica com diagnóstico tardio de doença de Behçet
2010	Belczak, SQ; Aun, R; Valentim, L; Sincos, IR; Nascimento, LD; Puech-Leão, P^1^.	3a	Hospital das Clínicas (HC), Universidade de São Paulo (USP), São Paulo, São Paulo, Brasil.	Tratamento endovascular de aneurismas da aorta em pacientes com doença de Behçet
2010	Laurenti, MR; Demartini Junior, Z; Santos, RMT; Spotti, AR^26^.	3b	Hospital de Base e Centro do Cérebro e Coluna, São José do Rio Preto, São Paulo, Brasil.	Tratamento endovascular de aneurisma dissecante da artéria vertebral em pacientes com doença de Behçet
2009	Kim, WH; Choi, D; Kim, JS; Ko, YG; Jang, Y; Shim, WH^20^.	2b	Faculdade de Medicina da Universidade Yonsei, Shinchon-dong, Seodaemun-gu, Seul, República da Coreia.	Eficácia e segurança do aneurisma endovascular tratamento em pacientes com doença vásculo-Behçet
2008	Meyer Neto, JGC; Assuf, S; Penna, GL; Morard, MRS^6^.	3b	Hospital Federal de Ipanema, Rio de Janeiro, Rio de Janeiro, Brasil.	Tratamento endovascular de aneurisma volumoso da artéria pulmonar em paciente portador de doença de Behçet
2007	Jung, NY; Kim, SK; Chung, EC; Park, H; Cho, YK^11^.	3b	Departamento de Medicina Interna, Kangbuk Samsung Hospital, Escola de Medicina da Universidade Sungkyunkwan, Seul, Coreia do Sul.	Tratamento endovascular para ruptura de aneurisma da artéria intra-hepática em paciente com síndrome de Behçet
2006	D’Alessandro, GS; Machietto, RF; Silva, SM; Campos Júnior, W; Akel, CJ; Etchebehere, RM; Cardoso, RM; Izukawa, NM^7^.	3b	Serviço de Angiologia e Cirurgia Vascular, Hospital Professor Edmundo Vasconcelos, São Paulo, São Paulo, Brasil.	Tratamento endovascular de aneurisma de artéria poplítea como manifestação da doença de Behçet descompensada
2006	Serratto, VA; Loyola Netto, JG; Yoshizumi, L; Paiva, E^12^.	3b	Serviço de Reumatologia do Departamento de Clínica Médica do Hospital de Clínicas da Universidade Federal do Paraná (UFPR), Curitiba, Paraná, Brasil.	Tratamento endovascular da doença de Behçet com extensa trombose venosa
2005	Alves, CMR^27^.	2a	Escola Paulista de Medicina, Universidade Federal de São Paulo, São Paulo, São Paulo, Brasil.	Tratamento endovascular em situações especiais: doenças do tecido conectivo, aortites não infecciosas, aneurismas micóticos, aneurisma isolado das artérias ilíacas e urgências
2005	Robazzi, TCMV; Arruti, R; Souza, AK; Santiago, MB^28^.	3b	Serviço de Reumatologia Pediátrica do Hospital São Rafael, Salvador, Bahia, Brasil.	Tratamento endovascular de doença de neuro-Behçet de início na infância
2004	Silistreli, E; Karabay, O; Erdal, C; Serbest, O; Guzeloglu, M; Çatalyurek, H; Açikel, U; Turkey, I^22^.	3b	Departamento de Cirurgia Cardiovascular, Universidade de DokuzEylül, Escola de Medicina, Izmir, Turquia.	Doença de Behçet: tratamento de pseudoaneurisma da artéria poplítea por stent endovascular e implantação de enxerto
2002	Albuquerque, PR; Terreri, MTRA; Len, CA; Hilário, MOE^17^.	2b	Escola Paulista de Medicina, São Paulo, São Paulo, Brasil.	Doença de Behçet na infância

## RESULTADOS E DISCUSSÃO

A DB acomete tanto o sistema venoso quanto o arterial, com uma incidência de 88% de tromboses venosas/tromboflebites e varizes, nos acometimentos venosos, e de 12% de oclusões/estenose, aneurismas e pseudoaneurismas^25^. No envolvimento vascular, as lesões arteriais são menos frequentes que as venosas, e as lesões arteriais são responsáveis por apenas 12% das complicações vasculares na síndrome de Behçet. As lesões arteriais geralmente se desenvolvem na aorta e na artéria pulmonar e em seus ramos principais^9^. O local mais comum de formação do aneurisma é a aorta abdominal, seguida pelas artérias pulmonar, femoral, subclávia, poplítea, carótida comum, coronária, braquial, ulnar, ilíaca comum, ilíaca externa, tibial, renal, cerebral, axilar e esplênica^9^, sendo 65% por degenerações aneurismáticas e 35% por doenças oclusivas^25^. A síndrome de Behçet é agora reconhecida como um distúrbio sistêmico com envolvimento mucocutâneo, oftálmico, neurológico, cardiovascular, pulmonar, gastrointestinal, urogenital e musculoesquelético. Qualquer artéria ou veia do corpo pode ser afetada, e o mento da árvore vascular manifesta-se patologicamente como oclusão arterial, aneurisma, oclusão venosa e varizes^9^. Mesmo que os aneurismas na DB não sejam muito prevalentes, a sua ruptura é a principal causa de morte. Essa ruptura relaciona-se com o processo inflamatório dos tecidos perianeurismáticos e das reações fibróticas^3^. Considera-se como opções terapêuticas os tratamentos já citados: clínico, cirúrgico aberto e via endovascular. Em relação ao tratamento endovascular especificamente, desde 1984, há um número crescente de relatos da eficácia dessa técnica no tratamento de aneurisma de aorta abdominal (AAA) em pacientes com DB^3^.

As vasculites sistêmicas são um grupo de doenças raras e heterogêneas de etiologia desconhecida, caracterizadas por inflamação e necrose nas paredes dos vasos. A prevalência da DB é alta nos países da Rota da Seda (ou seja, do Oriente Médio ao Extremo Oriente, incluindo Turquia, Irã, China e Japão) e nos países mediterrâneos^9^.

Até o momento, a prevalência e a incidência de vasculites sistêmicas não são conhecidas no Brasil. O país é grande e tem uma população heterogênea, que compreende origem portuguesa entre negros e indígenas, frequentemente misturados como mulatos^9^. Descendentes de italianos e alemães são frequentes nas regiões Sul e Sudeste do país, enquanto descendentes de japoneses, coreanos, judeus, libaneses e sírios, em geral, vivem no Sudeste do Brasil, especialmente no estado de São Paulo. Portanto, diferenças significativas quanto à epidemiologia das vasculites sistêmicas podem ser encontradas em todo o Brasil^9^.

O tratamento clínico com uso de corticoides e imunossupressores (ciclofosfamida e azatioprina)^3^ deve ser realizado antes de qualquer tratamento cirúrgico aberto ou endovascular, uma vez que há uma alta taxa de recorrência da doença aneurismática nos doentes com DB^14^. No entanto, quando não há remissão dos sintomas importantes e do risco de ruptura apenas com o tratamento clínico, o tratamento em regime de urgência é indicado^25^. Há, ainda, o uso adjuvante de corticoides ou imunossupressores no período pós-operatório, assim como o uso de anticoagulantes ou antiagregantes plaquetários, com o objetivo de reduzir o risco de oclusão do enxerto^16^.

O tratamento na DB visa prevenir danos irreversíveis que ocorrem principalmente no início do curso da doença, especialmente no grupo de alto risco, homens jovens, além de prevenir exacerbação do envolvimento mucocutâneo e articular, geralmente não causando danos, mas afetando a qualidade de vida. Qualquer paciente com DB e doença inflamatória ocular que afete o segmento posterior deve estar em regime de tratamento que inclua azatioprina e corticosteroides sistêmicos^29^. O envolvimento ocular na DB segue um curso remitente e recidivante, e os ataques inflamatórios recorrentes resultam em danos irreversíveis e perda visual. A supressão da inflamação e a prevenção de recorrências de ataques oculares devem ser os objetivos. Não há evidências firmes para orientar o manejo da doença dos principais vasos na DB^29^.

Para o tratamento da trombose venosa profunda aguda na DB, são recomendados agentes imunossupressores como corticosteroides, azatioprina, ciclofosfamida ou ciclosporina A. Para o manejo de aneurismas arteriais pulmonares e periféricos, são recomendados ciclofosfamida e corticosteroides^29^.

Os antagonistas do TNF-α e IFN têm demonstrado boa eficácia e são os agentes de primeira linha usados para melhorar o prognóstico da DB. O tratamento com antagonistas do TNF-α (infliximabe, etanercepte, adalimumabe) baseia-se no controle da resposta inflamatória e mostra-se eficiente em manifestações graves e refratárias da DB. A terapia com IFN, sobretudo o INF-α, tem demonstrado benefícios no tratamento habitual da doença, com atividades antiviral, antitumoral e imunomoduladora eficazes no manejo da DB^29^. No entanto, ainda há escassez de dados sobre essas abordagens terapêuticas ideais, e há falta de marcadores laboratoriais informativos para monitorar a progressão da doença^10^.

Os aneurismas micóticos possuem uma incidência de 1% de todos os aneurismas e são ocasionados por uma infecção bacteriana a distância. Para o tratamento desses falsos aneurismas, é contraindicada a colocação de enxertos intravasculares, mas a colocação de stents por cirurgia endovascular tem sido relatada com sucesso na literatura, por reduzir os riscos em comparação à cirurgia convencional, principalmente em topografia de aorta torácica ou de múltiplos segmentos^27^.

As artérias mais comumente afetadas são a aorta abdominal, seguida pelas artérias femorais e pulmonares, sendo os aneurismas intracranianos de rara incidência. Por outro lado, o tratamento cirúrgico é a primeira escolha para os aneurismas cerebrais rompidos associados à DB, sendo o tratamento endovascular uma alternativa razoável à cirurgia em ruptura perifericamente localizada, em forma fusiforme, dissecção de pseudoaneurismas e de circulação posterior^26^. As manifestações relacionadas ao SNC ocorrem em maior frequência nos adultos. Essa é a forma mais grave da doença, se manifestando de 2 a 6 anos após o início da sintomatologia, com prevalência em torno de 5 a 7% da população adulta^28^.

O tratamento endovascular, por meio do implante de endopróteses, stents recobertos ou embolização com molas, pode ser empregado em situações eletivas com anatomia favorável. Assim, o diagnóstico de complicações vasculares, principalmente de aneurismas, aliado ao tratamento correto, especialmente o endovascular, traz um melhor prognóstico para o paciente^24^. Dessa forma, a cirurgia endovascular passou a ser a via de preferência no tratamento cirúrgico nos casos de aneurismas arteriais, especialmente nos relacionados com DB^1^. O aneurisma arterial é o mais comum e constitui importante causa de morte na DB, secundária à sua ruptura. Por esse motivo, o tratamento cirúrgico é obrigatório e deve ser feito o mais precocemente possível^4^.

O melhor tratamento de aneurismas em pacientes portadores de DB ainda é controverso^1^. De forma geral, o tratamento na DB é variável de acordo com as manifestações clínicas do paciente e com suas gravidades^30^, mas o tratamento endovascular é uma alternativa relevante para os pacientes classificados como alto risco, uma vez que as taxas de morbidade e mortalidade (0,6-3,5%) após a cirurgia convencional ainda são altas^1^. Além disso, o reparo cirúrgico aberto tem um resultado menos satisfatório no que tange às complicações vasculares pós-operatórias, tais como pseudoaneurisma e oclusão de enxerto, sendo, assim, o método endovascular menos invasivo^1^. Ele possui menores chances de complicações no pós-operatório, com incidência de cerca de 19%^23^. Dessa forma, o tratamento endovascular vem se mostrando como uma alternativa promissora à cirurgia aberta no tratamento dos doentes^14^ e passou a ser a abordagem de escolha na maioria dos pacientes que possuem risco cirúrgico alto^23^.

O tratamento endovascular geralmente apresenta menor tempo operatório, de internação hospitalar e de perda de sangue se comparado ao tratamento cirúrgico aberto. Além disso, a taxa de sucesso ao liberar a prótese endovascular é alta (90% em pacientes que possuem baixo risco e 80% em pacientes que possuem risco entre moderado e alto). Por outro lado, o tratamento endovascular também possui limitações, principalmente relacionadas ao tamanho dos sistemas de entrega, aos vazamentos e à posição dos troncos principais^1^.

## CONCLUSÃO

Apesar de ainda não haver um consenso sobre o melhor tratamento de aneurismas vasculares para pacientes portadores da DB, a alternativa da cirurgia endovascular vem sendo a preferida em relação à cirurgia aberta de reparação, sobretudo em pacientes com alto risco cirúrgico, pelos seus benefícios em relação ao tempo intraoperatório, ao tempo de internação e à perda de sangue. Ademais, a terapia com corticoides e imunossupressores, como ciclofosfamida e azatioprina, é realizada de maneira adjuvante tanto no pré-operatório quanto no pós-operatório dos pacientes submetidos à correção de aneurisma decorrente da DB, independentemente da técnica escolhida.
